# Solid stress facilitates spheroid formation: potential involvement of hyaluronan

**DOI:** 10.1038/sj.bjc.6600158

**Published:** 2002-03-18

**Authors:** C Koike, T D McKee, A Pluen, S Ramanujan, K Burton, L L Munn, Y Boucher, R K Jain

**Affiliations:** Edwin L Steele Laboratory, Department of Radiation Oncology, Massachusetts General Hospital, 100 Blossom Street, Cox-7, Harvard Medical School, Boston, Massachusetts, MA 02114, USA; Division of Bioengineering and Environmental Health, Massachusetts Institute of Technology, Cambridge, Massachusetts, MA 02139, USA

**Keywords:** solid stress, spheroid, hyaluronan, agarose, Gompertz equation

## Abstract

When neoplastic cells grow in confined spaces *in vivo*, they exert a finite force on the surrounding tissue resulting in the generation of solid stress. By growing multicellular spheroids in agarose gels of defined mechanical properties, we have recently shown that solid stress inhibits the growth of spheroids and that this growth-inhibiting stress ranges from 45 to 120 mmHg. Here we show that solid stress facilitates the formation of spheroids in the highly metastatic Dunning R3327 rat prostate carcinoma AT3.1 cells, which predominantly do not grow as spheroids in free suspension. The maximum size and the growth rate of the resulting spheroids decreased with increasing stress. Relieving solid stress by enzymatic digestion of gels resulted in gradual loss of spheroidal morphology in 8 days. In contrast, the low metastatic variant AT2.1 cells, which grow as spheroids in free suspension as well as in the gels, maintained their spheroidal morphology even after stress removal. Histological examination revealed that most cells in AT2.1 spheroids are in close apposition whereas a regular matrix separates the cells in the AT3.1 gel spheroids. Staining with the hyaluronan binding protein revealed that the matrix between AT3.1 cells in agarose contained hyaluronan, while AT3.1 cells had negligible or no hyaluronan when grown in free suspension. Hyaluronan was found to be present in both free suspensions and agarose gel spheroids of AT2.1. We suggest that cell–cell adhesion may be adequate for spheroid formation, whereas solid stress may be required to form spheroids when cell–matrix adhesion is predominant. These findings have significant implications for tumour growth, invasion and metastasis.

*British Journal of Cancer* (2002) **86**, 947–953. DOI: 10.1038/sj/bjc/6600158 www.bjcancer.com

© 2002 Cancer Research UK

## 

The local microenvironment of neoplastic cells plays a critical role in tumour angiogenesis, growth, invasion and metastasis. With the availability of novel optical techniques, our understanding of the biochemical environment of tumours (e.g. hypoxia, low pH) has increased dramatically in recent years (Carmeliet and Jain, 2000; Helmlinger *et al*, 1997b, 2000). However, due to a lack of similar techniques for reproducing and measuring *in vivo* solid stress, the role of the mechanical environment in tumour biology is poorly understood.

By growing neoplastic cells as spheroids in agarose gels (Sutherland, 1988), we have recently measured the solid (mechanical) stress generated by these cells during growth (Helmlinger *et al*, 1997a). We have shown that this stress inhibits the growth of spheroids and that this growth-inhibiting stress is in the range of 45 to 120 mmHg. This stress may be responsible for the collapse of blood and lymphatic vessels in tumours (Jain, 1988; Griffon-Etienne *et al*, 1999; Leu *et al*, 2000), and may also influence tumour growth and metastasis (Fidler, 1991).

Here we test the hypothesis that solid stress can facilitate the formation of spheroids. We first screened for cell lines that do not form spheroids in free suspension. We then tested the possibility that the stress generated in an agarose gel would cause the cells to grow as spheroids. Moreover, if the stress is removed by treating these gels with agarose, we tested the likelihood that these spheroids would disintegrate into smaller aggregates or single cells. We show here that the highly metastatic Dunning prostate carcinoma line AT3.1 satisfies these criteria. Whereas the low metastatic line AT2.1 forms spheroids in both free suspension and in gels, the highly metastatic line AT-3.1 primarily forms loose cell aggregates when cultured in free suspension, with sparse spheroid formation. AT-3.1 cells form spheroids when grown in agarose gels. Interestingly, once the spheroids are formed, solid stress inhibits the growth of both AT3.1 and AT-2.1 spheroids.

## MATERIALS AND METHODS

### Culture of spheroids in free-suspension and agarose gels

Cell lines derived from the Dunning rat prostate carcinoma were obtained from Dr John Issacs, Johns Hopkins University (Baltimore, MD, USA) (AT2.1, AT3.1) and maintained as described previously (Isaacs *et al*, 1986). For suspension culture, cell culture dishes were coated with poly (2-hydroxyethylmethacrylate) (50 mg ml^−1^, Sigma Chemical Co., St. Louis, MO, USA). For the solid stress assay, gels of various agarose concentration (Type VII, Low Gelling Temperature, Sigma) were prepared. Gels seeded with single-cell suspensions at a density of 5×10^3^ cells ml^−1^ were prepared in sterile well inserts (1-inch outside diameter, Collaborative Biomedical Products, Bedford, MA, USA) with porous, 1 μm filter membranes. This membrane separated two medium compartments. Agarose stock solutions of 2.0% (w v^−1^) agarose were made with distilled water and autoclaved. Agarose gels were prepared to reach standard concentration of culture medium with 2.0% agarose stock, 10× concentration of culture medium and distilled water. Both free suspension and agarose cultures were maintained in RPMI supplemented with 10% foetal calf serum and dexamethasone. The culture medium was changed every other day.

Images of spheroids were obtained using a Sony video camera (DXC-970MD) attached to an Olympus microscope (BH2-UMA, Olympus, Melville, NY, USA) with a 10× objective under transmitted light. Images were analysed using an automated procedure written as a macro for NIH Image (version 1.61, available via download at http://rsb.info.nih.gov/nih-image). Fields for observation were chosen randomly, and the projected areas of all spheroids in a given field were recorded.

### Removal of agarose gel and suspension culture

Spheroids in agarose gels were treated with agarase (10 U ml^−1^, Sigma) for 2 days. Spheroids were separated from the degraded agarose gel by centrifugation (1000 r.p.m., 3 min), and resuspended with culture medium.

### Morphological study of spheroids under solid stress

Spheroids cultured in agarose gel for 10 days were fixed with 4% paraformaldehyde for 3 h, washed in PBS and then fixed in osmium (1%) for 2 h. The spheroids were then dehydrated in increasing concentrations of ethanol and embedded in Polybed 812. Sections with a thickness of 2 μm were stained with toluidine blue.

### Labelling of hyaluronan with the hyaluronan binding protein

AT-2.1 and AT-3.1 cells were grown in 1% agarose or in free suspension. After 3 weeks, spheroids were fixed in 4% paraformaldehyde. To facilitate handling, free suspension spheroids were concentrated by centrifugation and embedded in agarose. Sections 5 μm thick were prepared from paraffin blocks. The paraffin was removed, and the slides were washed with PBS between each step. The slides were incubated with the biotinylated hyaluronan binding protein (Calbiochem, CA, USA), then with streptavidin-coupled horseradish peroxidase), and finally with diaminobenzidine. Negative and positive controls ensured that the staining was specific for hyaluronan (data not shown). The slides were counterstained with haematoxylin and Scott's water, and then dehydrated and coverslipped.

### Gompertz analysis

The Gompertz equation is traditionally used to describe the size-limiting growth of tumours and tumour spheroids and is given by:


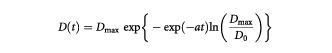


in which *D_0_* and *D(t)* are measures of spheroid size (either diameter or volume) at initial time and time *t* respectively, *D_max_* is the limiting size, and *a* is the specific growth rate (Winsor, 1932). The resulting growth curve has a sigmoidal shape and reflects a continuously increasing doubling time that causes an asymptotic approach to size *D_max_*.

The Gompertz equation was used to fit the spheroid growth curves of diameter *vs* time. Kaleidagraph software routines for least square error curve fitting were used to determine curve fits and parameter values with associated standard errors.

## RESULTS AND DISCUSSION

### Solid stress facilitates spheroid formation

**Figure 1:**
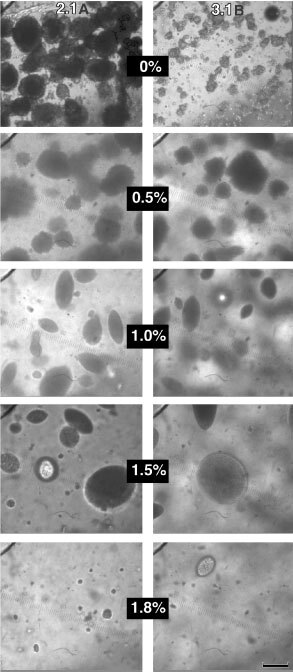
AT2.1 (**A**) and AT3.1 (**B**) cultures after 30 days in agarose gels. The left and right columns show AT2.1 and 3.1, respectively, in various concentrations of agarose (0% refers to cells in culture media). Note that at the highest concentration, 1.8%, cell growth is inhibited in both cell lines. Scale bar=100 μm

**Figure 2:**
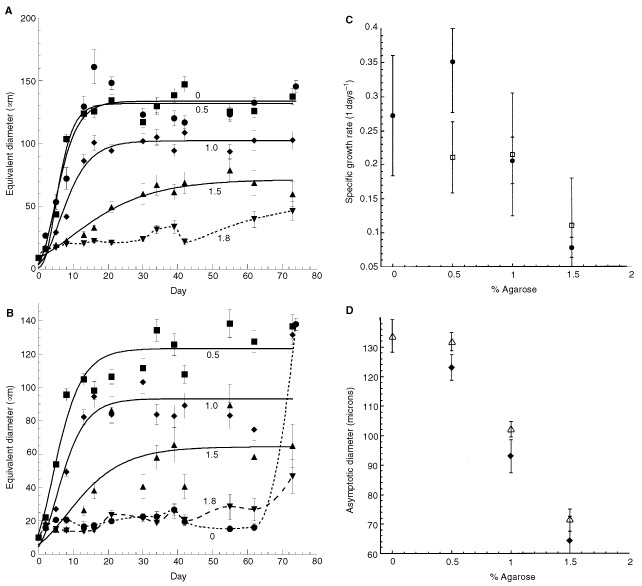
Growth curves and Gompertz fits for AT2.1 (**A**) and AT3.1 (**B**) spheroids in gels of various concentration. Data that could not be fit with the Gompertz equation are denoted with a dotted line. Agarose concentrations are indicated on the plot. The rapid rise at the last time point for the 0% sample in (**B**) is likely due to loose aggregation resulting from crowding of the system rather than actual spheroid formation. (**C**) and (**D**) give the Gompertz growth parameters α (specific growth rate) and D_max_ (asymptomatic size), respectively. (**C**) Solid circles represent AT2.1 and open boxes AT3.1. (**D**) Open triangles represent AT2.1 and solid diamonds AT3.1.

We defined spheroids as multicellular aggregates with spheroidal or ellipsoidal shapes. To determine whether solid stress can facilitate spheroid formation, we used a highly metastatic rat prostate carcinoma line AT3.1, which primarily forms loose cell aggregates when cultured in free suspension, with rare spheroid formation for up to 70–80 days in free suspension culture (Figure 1B

). As a comparison, we used the low metastatic variant AT2.1 which forms spheroids up to diameters of ∼150 μm after ∼2 weeks in free suspension (Figure 1A). When cultured in agarose gels, both cell lines formed spheroids (Figure 1). Surprisingly, the growth kinetics and maximum spheroid sizes were similar for both cell lines (Figure 2

). These results suggest that solid stress provided the necessary conditions to overcome the differences in aggregation/adhesion between the two cell lines, because both behaved similarly in gels. There are two possible mechanisms that could explain this: either the gel physically confines the cells, forcing them to adopt a spheroidal geometry, or the gel elicits a biological response that increases the adhesion between cells (or between the cells and matrix). This finding has important implications for the effect of host tissue stress on tumour compactness and metastasis.

The spheroids shown in Figure 1 illustrate an interesting observation made during the experiment: in agarose gels the spheroids become more ellipsoidal than in free suspension. This may be due anisotropic mechanical properties of the gel.

### Solid stress inhibits spheroid growth

To determine the effect of increasing solid stress on growth kinetics, we cultured both cell lines in gels of various concentration and measured the spheroid size as a function of time. We also fit the growth curves with the Gompertz equation. The model yields two parameters: the specific growth rate, *a* (day^−1^) and the maximum (asymptotic) spheroid diameter, *D_max_* (μm). As shown in Figure 2, increasing the gel concentration from 0.5 to 1.5% agarose decreases *D_max_* from ∼150 to ∼65 μm. In 1.8% agarose gels the growth is completely arrested. We suggest that this inhibition in growth results from increasing stress as trapped spheroids grow within denser gels, consistent with our previous data on five different cell lines: LS174T (human colon adenocarcinoma), MCaIV (murine mammary carcinoma), and the clones (A, B, C) of a rat rhabdomyosarcoma, BA-HAN-1 (Helmlinger *et al*, 1997a).

Unlike our previous study, we found that the specific growth rate, *a*, decreased with increasing gel concentration for AT2.1 (*P*=0.023), but not for AT3.1 (Figure 2C). Without knowing the cellular proliferation rates (e.g. PCNA staining), apoptosis rates (e.g. TUNEL staining) and cell densities (e.g. PI staining), it is difficult to determine the mechanisms underlying this difference. In parallel shorter-term experiments, cells were seeded in agarose gels at both higher (up to 20×10^3^ cells ml^−1^) and lower (2.5×10^3^ cells ml^−1^) seeding densities, and corresponding growth curves were also fit with the Gompertz equation. Although the limiting size *D_max_* could not be determined accurately for these experiments due to their shorter duration, spheroids grown in gels with low seeding densities reached sizes of 300 μm (AT2.1) and 250 μm (AT3.1) by day 30, exceeding maximum measurements at other seeding densities, even at longer times. Accurate estimation of the growth rate *a*, which is determined largely by the rapid growth phase and not the long-term data, was possible and showed no significant variation with seeding density at any gel concentration. Thus, we note that seeding density can affect asymptotic spheroid size but does not appear to affect growth rate (data not shown). We speculate that this result is due to solid stress: stress increases more rapidly in gels containing a high density of (growing) spheroids which thus limits their growth.

### Relieving stress causes loss of spheroid integrity

**Figure 3:**
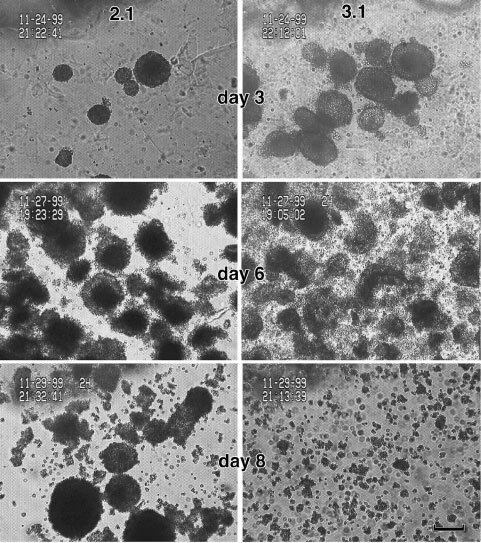
Enzymatic digestion of agarose gel cultures after 30 days of growth. AT2.1 and AT3.1 are shown in the left and right columns, respectively. Images shown are from 3, 6, and 8 days after release of spheroids from gel. Note that the AT3.1 cells return to a dis-aggregated state, but the AT2.1 maintain their spheroidal morphology at 8 days after release. Scale bar=100 μm.

When stress around plateau–phase spheroids was relieved by treatment of the gel with agarase, the two cell lines responded differently. Similar to our previous findings (Helmlinger *et al*, 1997a), the AT2.1 spheroids retained their morphology and resumed their growth as in free suspension (Figure 3

). The AT3.1 cells also retained their morphology for the first several days, but lost their integrity by days 6–8 and disintegrated into a loose collection of cells. The latter were reminiscent of cellular aggregates in free suspension. However, the spheroids did not lose their integrity as rapidly upon treatment with agarase as would be expected for unassociated cells held in proximity only by gel. Evidence for this comes from the fact that pelleting by centrifugation did not contribute to aggregation, and the pelleted cells were readily separated (data not shown). These results again support the hypothesis that solid stress facilitates spheroid formation by inducing some form of adhesion between cells.

### Morphological differences may result from the stress response

**Figure 4:**
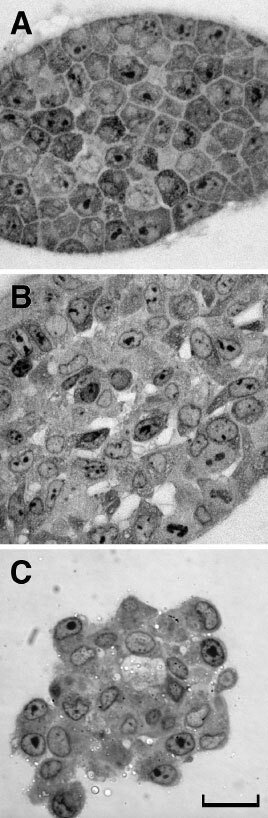
Histological sections of spheroids in agarose gels. AT3.1 cells in agarose, are loosely-packed with a uniform matrix space observed between the cells (**A**). On the other hand, in spheroids of AT2.1 (**B**) and aggregates of AT3.1 (**C**) there is no visible matrix between most of the cells. Scale bar=20 μm.

To gain insight into the differential behaviour of AT2.1 and AT3.1 cell lines with respect to spheroid formation, we prepared histological sections of spheroids. As seen in Figure 4

, the cells of AT3.1 spheroids in agarose gels have polygonal shapes and are separated by uniform spaces with a regular width (0.45–0.8 μm) occupied by the extracellular matrix (Figure 4A). On the other hand, there appears to be (at least at the light microscopic level) no discernible spacing between most of the cells in AT2.1 spheroids in agarose (Figure 4B), and AT2.1 and AT3.1 cells in free suspension (Figure 4C). Large extracellular spaces are observed occasionally in AT2.1 spheroids and in free suspensions of 2.1 spheroids and 3.1 aggregates (Figure 4B,C). Spheroid formation by AT3.1 cells growing in agarose could result solely from the stiffness (or the limited compliance) of the agarose gel which would compact the cells and the matrix. Alternatively, cell–matrix adhesion may also contribute to spheroid formation in AT3.1. This is supported by the observations that (a) AT3.1 cells are closely apposed in free suspension aggregates but separated by matrix in agarose gels (Figure 4A,C), and (b) the adhesion holding together the large spheroids is not immediately reversed upon removal from the gel. Although formation and compaction of spheroids can be mediated by E-cadherin (Kantak and Kramer, 1998; St. Croix *et al*, 1998) it has been shown that AT2.1 and 3.1 cell lines do not express E-cadherin (Bussemakers *et al*, 1992). Hyaluronan may also be important in spheroid formation, as hyaluronan participates in cell–cell adhesion and cellular aggregation of transformed cells (Underhill and Toole, 1981), and it has been shown to influence both the formation and compactness of spheroids by tumour cell lines (St. Croix *et al*, 1996, 1998). The addition of hyaluronidase to compact (tight) spheroids induced spheroids with a loose morphology (St. Croix *et al*, 1996). Also, treatment of tumour cells with hyaluronidase can prevent the formation of cellular aggregates and spheroids (St. Croix *et al*, 1998; Mueller *et al*, 2000).

### Solid stress induces hyaluronan synthesis

**Figure 5:**
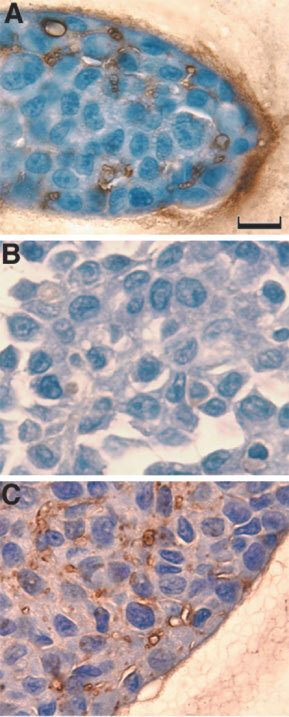
Spheroid sections in gels and in free suspension stained with the hyaluronan binding protein. Spheroids of AT3.1 grown in 1% agarose gels (**A**), AT3.1 aggregates in free suspension (**B**) and AT2.1 spheroids in 1% agarose gels (**C**). Brown staining indicates the presence of hyaluronan, while nucleii are stained blue (haematoxylin). In AT3.1 spheroids in agarose (**A**), hyaluronan staining is localised between the cells and at the spheroid–agarose interface. In contrast, the hyaluronan staining is not observed in free suspension aggregates of AT3.1 (**B**). Hyaluronan is associated with the surface of tumour cells or appears as globular structures in AT2.1 spheroids (**C**). Scale bar=25 μm.

To test if hyaluronan was associated with spheroid formation in AT3.1 cells in agarose, the hyaluronan binding protein was used to localise hyaluronan in histological sections. In AT3.1 spheroids, hyaluronan staining was found between tumour cells and at the spheroid–agarose interface (Figure 5A

). Hyaluronan staining was absent or rarely detected in AT3.1 aggregates grown in free suspension (Figure 5B). AT2.1 spheroids expressed hyaluronan both when grown in free suspension and under solid stress conditions, the staining was found lining the surface of tumour cells or appeared as globular structures of varying sizes between tumour cells (Figure 5C). The increased expression of hyaluronan in AT3.1 spheroids, indicates that solid stress increases the synthesis of hyaluronan. The effect of a mechanical stimuli on hyaluronan synthesis by other cell types is variable. Mechanical strain can increase the synthesis of hyaluronan by embryonic fibrocartilage cells (Dowthwaithe *et al*, 1999), whereas the static compression of cartilage does not modify the synthesis of hyaluronan (Kim *et al*, 1996). The presence of hyaluronan between cells of AT3.1 spheroids and functional studies using hyaluronidase, mentioned previously (St. Croix *et al*, 1998; Mueller *et al*, 2000), suggest that hyaluronan may be necessary for cell-cell adhesion and spheroid formation by AT3.1 cells. The slow disintegration rate of AT3.1 spheroids following release from solid stress conditions supports this conclusion, as the gradual degradation of the hyaluronan present at the edge of spheroids or between cells could occur at a slow rate. The half-life of hyaluronan of the skin and eye can vary between 1–4.5 days and 10–30 days, respectively (Fraser and Laurent, 1998). The relative importance of cell–cell adhesion and matrix deposition/adhesion in mediating spheroid formation requires further investigation.

In summary, solid stress facilitates the formation of spheroids from cells that exhibit little homotypic adhesion in free suspension, and release of this stress causes loss of spheroid integrity. Solid stress can increase hyaluronan synthesis by tumour cells.
